# Supplementary material for: Thermal Inactivation of Hepatitis E Virus in Pork Products Estimated with a Semiquantitative Infectivity Assay
Source: Microorganisms. 2023 Sep 29;11(10):2451. doi: 10.3390/microorganisms11102451 (PMC10609450; doi:10.3390/microorganisms11102451)
Supplement: Supplementary file 1 [file microorganisms-11-02451-s001.zip › Supplementary Figures S1-S5 and Tables S1-S7 with caption-Stunnenberg et al.pdf]

Supplementary figures

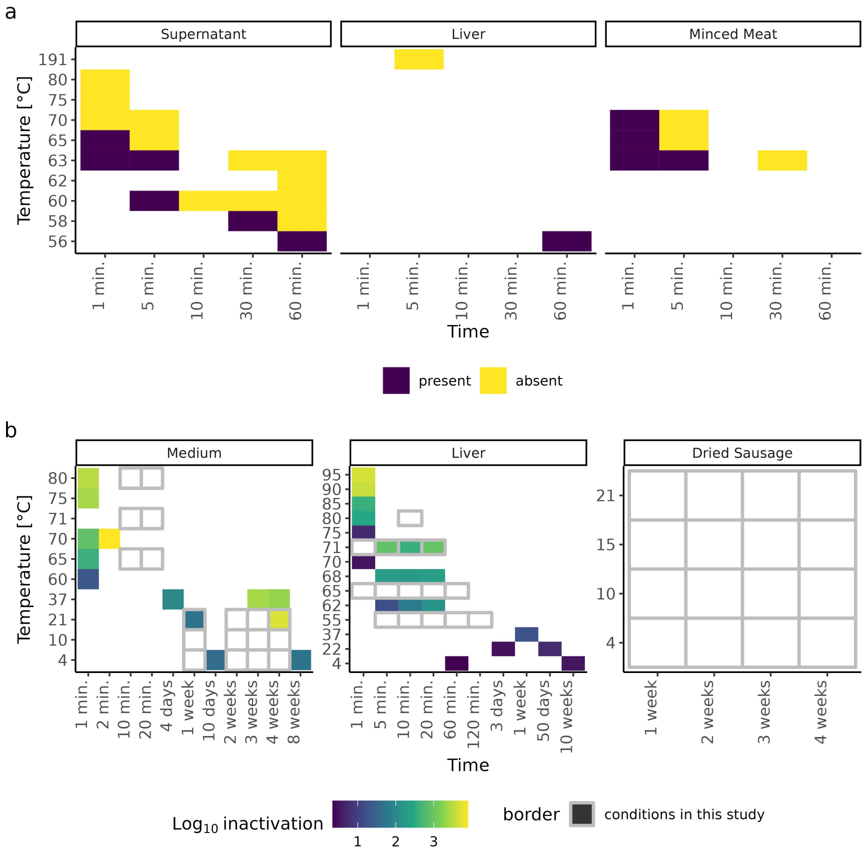

Figure S1. Heat map of available data in literature on HEV genotype 3 inactivation. (a) HEV-3 inactivation data from different studies [23, 24] after thermal treatment for different durations in cell culture medium, liver and minced meat. Presence and absence of HEV RNA was indicated by purple and yellow, respectively; (b) Log10 HEV-3 inactivation data from different studies after thermal treatment for different durations. Detection of infectious HEV in cell culture medium, using a cell culture method, were assessed [26] as well as the detection of HEV RNA in liver matrices [25, 27]. Quantitative data ranged from full (> 4 log) inactivation (yellow) to no inactivation (purple). Conditions boxed in grey are conditions tested in the current study.

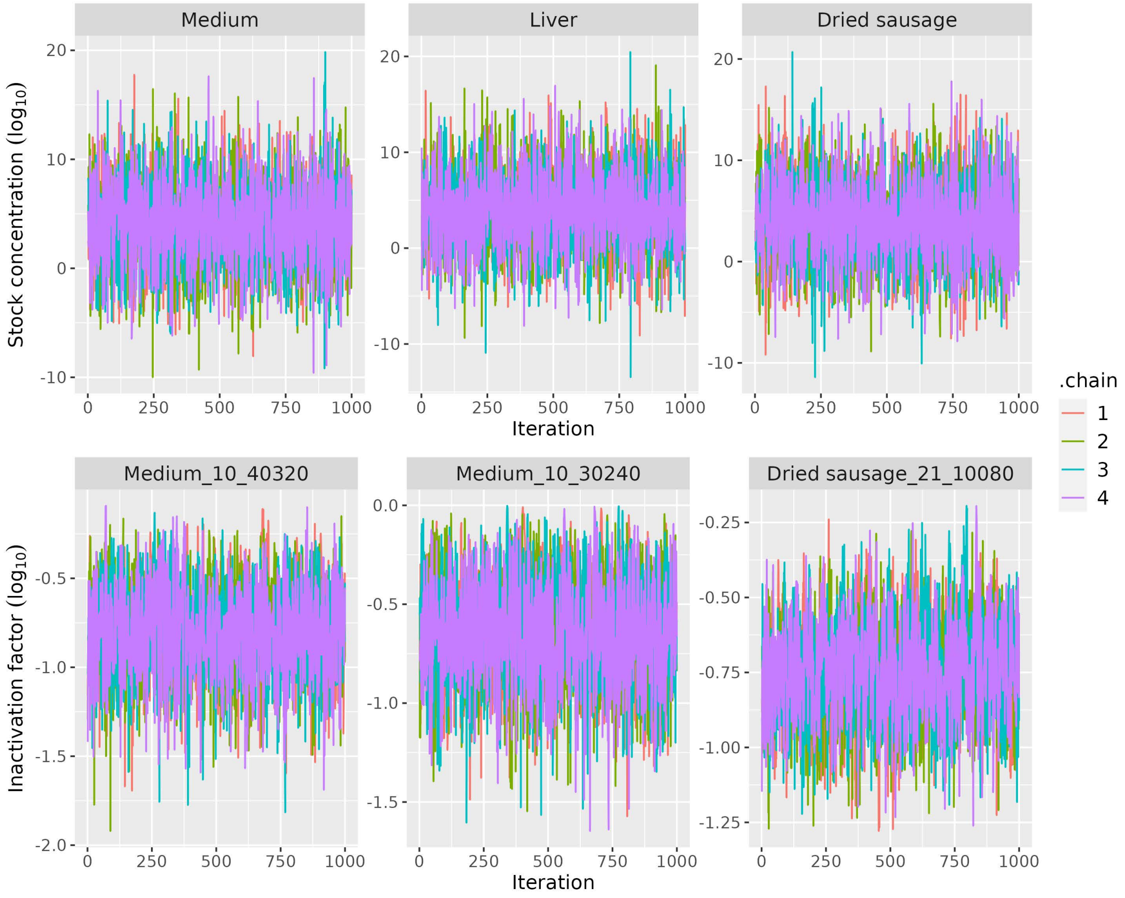

Figure S2. Traceplots of a few selected parameters. The x-axis shows the iterations of the Markov chains, and the y-axis shows samples from the posterior distributions at this iteration. Four chains were run in parallel. The figures show that the chains are stable, indicating reliable convergence to a posterior estimate.

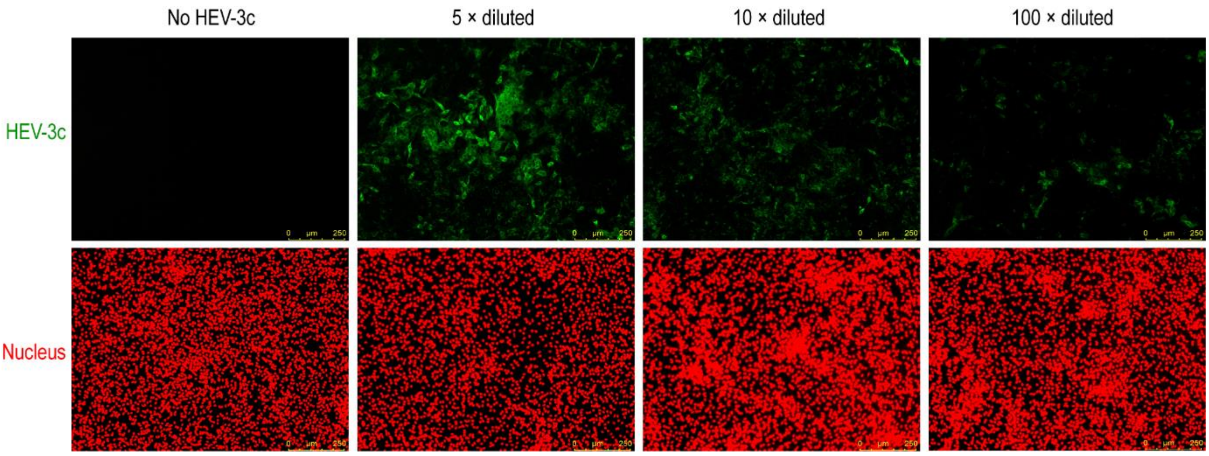

Figure S3. Cell culture method for the detection of infectious HEV in medium. A549/D3 cells were either left untreated or were inoculated with HEV-3c in different concentrations (5, 10 and 100x diluted). After 14 days, HEV-3c capsid protein was detected with a FITC-conjugated antibody (shown in green), while DAPI (shown in red) was used to visualize the cell nucleus. Data is representative of four different wells containing A549/D3 cells that were infected and examined with immune fluorescence.

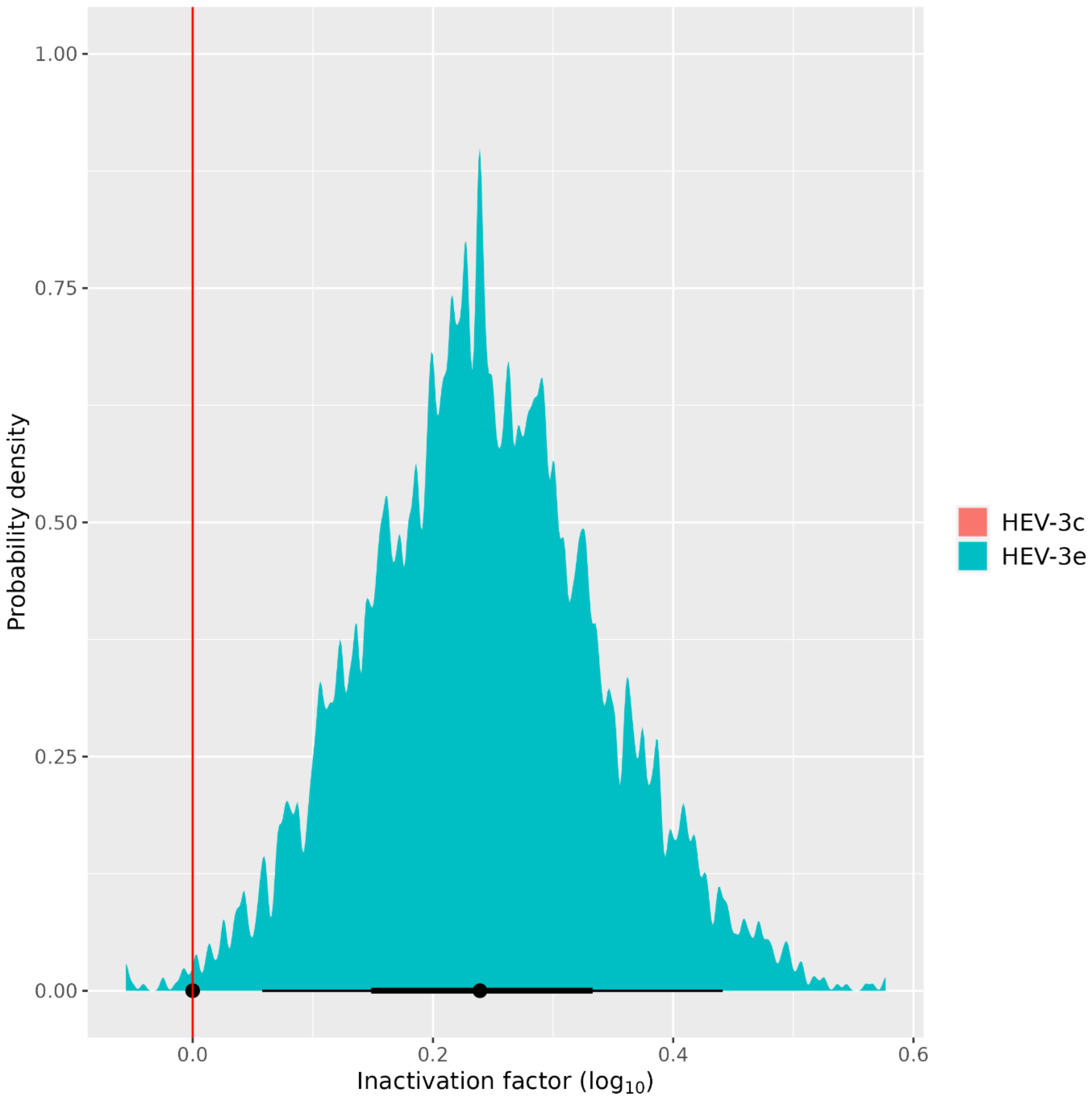

Figure S4. The difference in HEV-3c and HEV-3e inactivation factors (log_{10}). Inactivation factors per HEV-3 subtype were estimated using the Bayesian MPN-method and were based on the combined data for all time-temperature combinations tested in culture medium, dried sausage and liver homogenate experiments. The red line indicates HEV-3c, which was set at 0. HEV-3e shows less inactivation as compared to HEV-3c (inactivation factor of ± 0.25 log_{10}). Black lines indicate 50% and 95% confidence intervals.

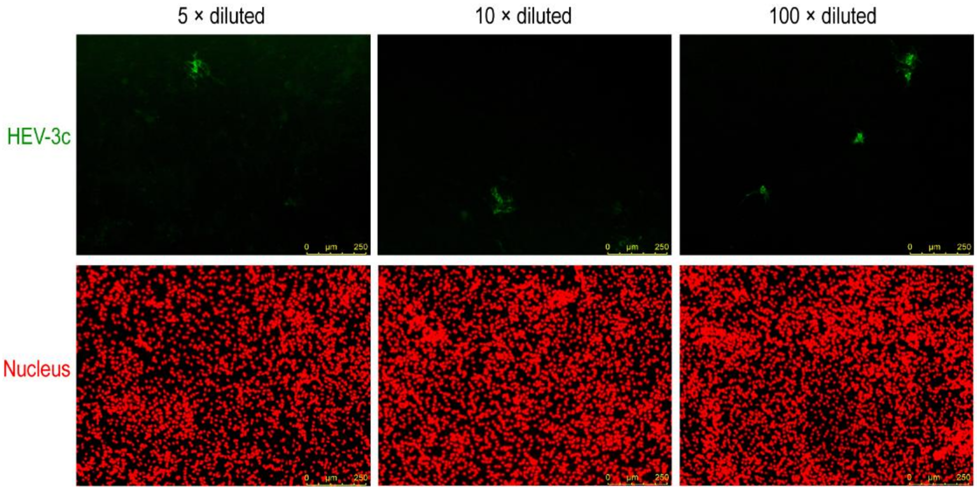

Figure S5. The detection of infectious HEV isolated from naturally-infected pig livers. A549/D3 cells were inoculated with HEV isolated from pig livers, in different concentrations (5, 10 and 100x diluted). After 14 days, HEV capsid protein was detected with a FITC-conjugated antibody (shown in green), while DAPI (shown in red) was used to visualize the cell nucleus. Data is representative of 1 pig liver (L-07), with eight different wells with A549/D3 cells per dilution, and examined with immune fluorescence.

Supplementary tables

Table S1. Estimated concentrations of infectious HEV-3c and HEV-3e stocks. Propagation of HEV in PLC/PRF/5 cells to obtain HEV-3e was performed twice (stock 1 and stock 2). One stock was prepared for experiments performed with HEV-3c only and therefore data on stock 2 is not available (n.a.). Concentrations were estimated using the Bayesian MPN-method and are given in average $\log_{10}$ viral particles/mL with the corresponding 95% Bayesian confidence interval (C.I.).

Matrix	HEV-3 subtype	Stock 1 Log_{10} viral particles/mL [95% C.I.]	Stock 2 Log_{10} viral particles/mL [95% C.I.]
Medium	3c	6.0 [5.8, 6.1]	n.a.
Medium	3e	6.1 [5.9, 6.2]	4.3 [4.1, 4.5]
Dried sausage	3c	5.1 [4.9, 5.3]	n.a.
Dried sausage	3e	4.7 [4.5, 4.9]	2.7 [2.5, 2.9]
Liver homogenate	3c	4.7 [4.5, 4.8]	n.a.
Liver homogenate	3e	5.0 [4.8, 5.2]	4.1 [3.8, 4.4]

Table S2. HEV-3c/e inactivation factor ($\log_{10}$) of virus in cell culture medium. This was assessed using the Bayesian MPN-method and posterior inactivation estimates are shown as a function of temperature and time. Data is representative of experiments performed with HEV-3c and HEV-3e. 95% confidence intervals (C.I.) are shown.

Temperature (°C)	Duration	HEV-3c/e inactivation factor ($\log_{10}$) [95% C.I.]
4	1 week	0.20 [-0.22, 0.72]
4	2 weeks	0.06 [-0.29, 0.54]
4	3 weeks	0.06 [-0.28, 0.54]
4	4 weeks	0.36 [-0.14, 0.94]
10	1 week	0.20 [-0.23, 0.73]
10	2 weeks	0.06 [-0.28, 0.55]
10	3 weeks	0.38 [-0.11, 0.94]
10	4 weeks	0.54 [0.07, 1.06]
21	1 week	0.67 [0.31, 1.08]
21	2 weeks	1.55 [1.19, 1.97]
21	3 weeks	1.22 [0.76, 1.69]
21	4 weeks	3.29 [2.94, 3.70]
65	10 min.	1.86 [1.40, 2.34]
65	20 min.	3.62 [3.23, 4.03]
71	10 min.	> 3.32[1]
71	20 min.	> 3.36[1]
80	10 min.	> 3.36[1]
80	20 min.	> 3.32[1]

[1]Experimental outcomes of all negative IF scores for all dilutions tested (absence of infectious HEV) contain very little information on the inactivation and are thus manifested as very high inactivation rates with very broad confidence intervals (Figure 1). Therefore, HEV-3c/e inactivation was estimated with 95 % certainty to be beyond the indicated HEV-3c/e inactivation factor ($\log_{10}$).

Table S3. IF results for A549/D3 cells inoculated with HEV-3c or HEV-3e-containing cell culture medium. Results are expressed as dilution for which all IF signals were scored 'negative', and are shown as a function of temperature, time and HEV-3 subtypes (HEV-3c and HEV-3e). The minimum number of wells tested at the indicated dilution are indicated between brackets. Conditions that were not available are indicated as n.a.

Temperature (°C)	Duration	Dilution with negative IF result for HEV-3c	Dilution with negative IF result for HEV-3e
-	0 min.	1000000 (6)	1000000 (6)
4	1 week	n.a.	10000 (4)
4	2 weeks	n.a.	10000 (4)
4	3 weeks	n.a.	10000 (4)
4	4 weeks	n.a.	1000 (4)
10	1 week	n.a.	10000 (4)
10	2 weeks	n.a.	10000 (4)
10	3 weeks	n.a.	1000 (4)
10	4 weeks	n.a.	10000 (4)
21	1 week	100000 (4)	1000000 (4)
21	2 weeks	n.a.	100 (4)
21	3 weeks	n.a.	100 (4)
21	4 weeks	100 (4)	100 (4)
65	10 min.	10000 (4)	10000 (4)
65	20 min.	5 (4)	1000 (4)
71	10 min.	n.a.	1 (4)
71	20 min.	n.a.	1 (4)
80	10 min.	n.a.	1 (4)
80	20 min.	n.a.	1 (4)

Table S4. HEV-3c/e inactivation factor (log10) of virus isolated from dried sausage. This was assessed using the Bayesian MPN-method and posterior inactivation estimates are shown as a function of temperature and time. Data is representative of experiments performed with HEV-3c and HEV-3e. 95% confidence intervals (C.I.) are shown.

Temperature (°C)	Duration	HEV-3c/e inactivation factor (log10) [95% C.I.]
4	1 week	0.53 [0.17, 0.93]
4	2 weeks	0.14 [-0.21, 0.55]
4	3 weeks	0.51 [0.13, 0.92]
4	4 weeks	1.20 [0.84, 1.60]
10	1 week	0.63 [0.25, 1.05]
10	2 weeks	0.83 [0.46, 1.23]
10	3 weeks	0.70 [0.31, 1.13]
10	4 weeks	0.86 [0.49, 1.26]
15	1 week	0.33 [-0.09, 0.78]
15	2 weeks	0.67 [0.21, 1.17]
15	3 weeks	0.67 [0.20, 1.16]
15	4 weeks	1.45 [0.99, 1.94]
21	1 week	0.60 [0.21, 1.01]
21	2 weeks	1.09 [0.69, 1.54]
21	3 weeks	1.39 [0.96, 1.85]
21	4 weeks	2.35 [1.99, 2.75]

Table S5. IF results for A549/D3 cells exposed to extracts of HEV-3c or HEV-3e-inoculated dried sausage. Results are expressed as dilution for which all IF signals were scored 'negative', and are shown as a function of temperature, time and HEV-3 subtypes (HEV-3c and HEV-3e). The minimum number of wells tested at the indicated dilution are indicated between brackets.

Temperature (°C)	Duration	Dilution with negative IF result for HEV-3c	Dilution with negative IF result for HEV-3e
-	0 min.	100000 (16)	100000 (16)
4	1 week	100000 (4)	100000 (4)
4	2 weeks	100000 (4)	10000 (4)
4	3 weeks	10000 (4)	10000 (4)
4	4 weeks	1000 (4)	1000 (4)
10	1 week	1000 (4)	100000 (4)
10	2 weeks	10000 (4)	10000 (4)
10	3 weeks	10000 (4)	10000 (4)
10	4 weeks	10000 (4)	1000 (4)
15	1 week	100000 (4)	10000 (4)
15	2 weeks	10000 (4)	10000 (4)
15	3 weeks	10000 (4)	10000 (4)
15	4 weeks	1000 (4)	1000 (4)
21	1 week	10000 (4)	10000 (4)
21	2 weeks	10000 (4)	1000 (4)
21	3 weeks	1000 (4)	1000 (4)
21	4 weeks	1000 (4)	100 (4)

Table S6. HEV-3c/e inactivation factor (log_{10}) of virus extracted from liver homogenate. This was assessed using the Bayesian MPN-method and posterior inactivation estimates are shown as a function of temperature and time. Data is representative of experiments performed with HEV-3c and HEV-3e. 95% confidence intervals (C.I.) are shown.

Temperature (°C)	Duration	HEV-3c/e inactivation factor (log_{10}) [95% C.I.]
55	5 min.	0.52 [0.06, 1.01]
55	10 min.	1.31 [0.85, 1.79]
55	20 min.	1.30 [0.87, 1.72]
55	60 min.	1.98 [1.59, 2.38]
55	120 min.	1.98 [1.55, 2.39]
65	1 min.	0.03 [-0.31, 0.40]
65	5 min.	1.99 [1.58, 2.40]
65	10 min.	2.55 [2.13, 3.00]
65	20 min.	2.97 [2.49, 3.50]
65	60 min.	3.56 [2.92, 4.42]
71	1 min.	0.78 [0.33, 1.25]
71	5 min.	> 3.69[1]
71	10 min.	> 3.71[1]
71	20 min.	> 3.72[1]
80	10 min.	> 2.88[1]

[1]Experimental outcomes of all negative IF scores for all dilutions tested (absence of infectious HEV) contain very little information on the inactivation and are thus manifested as very high inactivation rates with very broad confidence intervals (Figure 3). Therefore, HEV-3c/e inactivation was estimated with 95 % certainty to be beyond the indicated HEV-3c/e inactivation factor (log_{10}).

Table S7. IF results for A549/D3 cells exposed to extracts of HEV-3c or HEV-3e-inoculated liver homogenate. Results are expressed as dilution for which all IF signals were scored 'negative', and are shown as a function of temperature, time and HEV-3 subtypes (HEV-3c and HEV-3e). The minimum number of wells tested at the indicated dilution are indicated between brackets. Conditions that were not available are indicated as n.a.

Temperature (°C)	Duration	Dilution with negative IF result for HEV-3c	Dilution with negative IF result for HEV-3e
-	0 min.	100000 (18)	100000 (18)
55	5 min.	10000 (4)	10000 (4)
55	10 min.	100 (4)	10000 (4)
55	20 min.	10000 (4)	10000 (4)
55	60 min.	1000 (4)	1000 (4)
55	120 min.	100 (4)	1000 (4)
65	1 min.	100000 (4)	10000 (4)
65	5 min.	100 (4)	1000 (4)
65	10 min.	10 (4)	100 (4)
65	20 min.	10 (4)	10 (4)
65	60 min.	5 (4)	10 (4)
71	1 min.	1000 (4)	10000 (4)
71	5 min.	5 (4)	5 (4)
71	10 min.	5 (4)	5 (4)
71	20 min.	5 (4)	5 (4)
80	10 min.	n.a.	5 (4)
